# The Applicability of a Complete Archive of Keyhole Imagery for Land-Use Change Detection in China (1960–1984)

**DOI:** 10.3390/s25103147

**Published:** 2025-05-16

**Authors:** Hao Li, Tao Wang, Jinyu Sun

**Affiliations:** 1 School of Geography and Environment, Liaocheng University, Liaocheng 252059, China; lihao@lcu.edu.cn (H.L.); 2022401915@stu.lcu.edu.cn (T.W.); 2 Liaocheng Innovative High Resolution Data Technology Co., Liaocheng 252059, China; 3 Northeast Institute of Geography and Agroecology, Chinese Academy of Sciences, Changchun 130102, China

**Keywords:** Keyhole imagery, 1960s~1980s, land-use change, resolution, spatial distribution

## Abstract

Declassified Keyhole imagery partially provides multi-temporal coverage that can support land-use change analysis. However, the volume of commercial (paid) Keyhole data is much larger than that of free imagery, and the extent to which commercial data can enhance the application of Keyhole imagery for land-use change analysis remains unknown. In this work, the full archive of Keyhole images for China was obtained from the USGS to identify regions with repeated coverage automatically by using the ArcPy library in Python. The years from 1960 to 1984 were divided into five 5-year periods (T1, 1960~1964; T2, 1965~1969; T3, 1970~1974; T4, 1975~1979; and T5, 1980~1984). The Keyhole images’ metadata, including resolution, acquisition time, and image extent, were utilized to classify the images into meter level (C1), five-meter level (C2), and ten-meter level (C3). The spatial distributions of combinations of imagery at different resolutions for each period and the repeated coverage of imagery at each resolution across the five periods were investigated to extract repeated-coverage regions. The coverage proportions were nearly 100% for C1 imagery for the T3, T4, and T5 periods; C2 for T1 and T2; and C3 for T1 and T3. The T3 period featured extensive coverage at all three resolutions (66%). The T1 period was mainly covered by C2/C3 (93%), and T4 had C1/C3 coverage (68%). In contrast, T2 relied primarily on C2 imagery (100%), and T5 was only covered by C1 (96%). For C1 imagery, land-use changes in almost all areas in China in the T3/T4/T5 time span could be detected, and for C2 and C3 images, the corresponding time spans were T1/T2 and T1/T3. Although this study focused on repeated-coverage area detection within China, the methodology and Python codes provided allow for the implementation of an automated process for land-use change detection from the 1960s to the 1980s in other regions worldwide.

## 1. Introduction

Datasets that capture land uses over time intervals are critical to measuring and analyzing the dynamics of land-use changes [1]. With its consistent data collection and wide-ranging spatial scope, advanced remote sensing technology has become an essential instrument for analyzing land-use dynamics [2]. Currently, the most comprehensive global land-use datasets and their derived products predominantly rely on Landsat imagery [3]. Nevertheless, the lower spatial resolution (60–78 m) of early Landsat imagery undermined the reliability of land-use classification [4], leading to a gap in global land-use datasets for the 1970s and early 1980s. Therefore, exploring pre-modern remote sensing imagery and analyzing historical land-use status are crucial to enriching land-use change research, as well as highlighting the environmental impacts and driving forces of land use.

In the 1960s, the United States began launching a sequence of reconnaissance satellites that produced the so-called Keyhole (KH) imagery collection [5]. The primary objective of these missions was to surveil Soviet missile capabilities and China’s nuclear development initiatives [6]. However, the satellites also provided extensive global coverage, with a particular focus on regions such as Eastern Europe and Asia. The approximately 860,000 images captured with KH 1~6 between 1960 and 1972 and the 50,000 images collected with KH-7 and KH-9 between 1963 and 1984 were declassified and released to the public in 1995 and 2002 [7], respectively. The notable strengths of Keyhole imagery, such as its exceptional ground resolution (up to 0.6 m), worldwide coverage, and availability before the advent of modern remote sensing technologies [8], have established it as an indispensable resource for studies on land-use change and related fields from the 1960s to the present. Since 2020, the amount of research utilizing Keyhole imagery for land-use change analysis has already exceeded the volume of studies conducted between 2000 and 2020 [9]. For most multi-temporal research, Keyhole imagery has remained the earliest dataset in the time series [10,11]. It was initially applied for the identification of individual archeological features, like road networks in the Middle East [12] and glacier changes in the Himalayas and Antarctica [13]. Currently, Keyhole imagery-related research is available for Asia, Europe, Africa [14], and the Americas [15]. Developing countries often lack sufficient historical aerial imagery or maps recording land-use status in the past, or existing historical graphs remain undigitized and are not readily accessible [16]. Hence, Keyhole imagery could offer greater potential in developing countries, which account for over 70% of the global land area [17]. Ancient hidden cultural relics or tectonic geomorphology, often lost in modern landscapes due to current land uses and cover, could be detected by using Keyhole imagery [18], a record of land-cover characteristics before urban expansion in developing countries. The extended temporal coverage of the land surface provided by Keyhole imagery has enhanced our understanding of the driving forces of land-use change. Nister et al. [19] applied KH-4B imagery to analyze the conversion of farmland into forest in Budapest since the 1960s, demonstrating that land abandonment in this region emerged in the 1980s, earlier than the traditionally perceived period of the Soviet Union’s collapse in the 1990s. Since Keyhole imagery is composed of single grayscale-band images, spectral classification approaches often achieve a low accuracy [20], and most current research relies on visual interpretation to classify land-use status. However, the inefficiency of visual interpretation in Keyhole imagery has led more researchers to try conventional neural networks to identify point features and machine learning technologies to improve the precision of land-use status classification [21,22].

Keyhole imagery has shown its potential to broaden the time span of land-use change detection and deepen insights into the factors driving these changes. However, the authors of the existing studies leveraging Keyhole imagery frequently operate under the assumption that land cover remained constant from the 1960s to the 1980s [23], failing to account for the persistent land-use alterations that occurred throughout this period. Methodologically, most studies have depended on single-date Keyhole imagery to derive land-use data, which are then compared with information from subsequent remote sensing platforms, such as ETM or SPOT [24]. However, there is a lack of research employing multi-temporal Keyhole imagery to capture and compare land-use statuses at multiple time periods between the 1960s and 1980s. Different from modern satellites with regular revisit periods like TM and SPOT, those of the Keyhole missions were film-return satellites [25], with strong variability in spatial coverage. Although the rate of land-use change several decades ago may have been slower than that in recent decades [26], it is likely that land cover underwent transformations during that period. Even if only a limited number of areas experienced changes, these shifts could still shed light on the diverse drivers of land-use change across different stages of societal development [27]. Consequently, the assumption that land use remained static for over two decades (from the 1960s to the 1980s) could impede a thorough understanding of early land-use change processes and their underlying factors. Considering the dynamics of land-use changes and processing costs, global or regional land-use datasets are generally updated periodically (e.g., every five years). Consequently, related research studies commonly include the analysis of land-use changes’ corresponding driving factors over these intervals [28]. Hence, exploring the potential of Keyhole imagery for capturing such changes in the 1960s and 1980s is significant for the further application of this resource.

With an average spatial coverage frequency exceeding nine passes in China from 1960 to 1984, declassified Keyhole imagery appears capable of fulfilling the multi-temporal dataset requirements for land-use change research [29]. By investigating the repeated coverage provided by freely downloaded Keyhole imagery, Li H et al. [30] demonstrated that land-use change detection could only be conducted on around 30% of the total area in China. Since declassified Keyhole imagery includes free and pay-to-download collections, determining the usefulness of full-archive imagery for land-use change detection is crucial to understanding and enhancing Keyhole imagery’s potential in earth science. Therefore, in this research study, we analyzed the spatial distribution of full-archive Keyhole imagery at five-year intervals between 1960 and 1984. Due to the varying resolution of Keyhole images, they were classified into three categories, i.e., meter level (C1), five-meter level (C2), and ten-meter level (C3), based on their metadata. The period from 1960 to 1984 was divided into five intervals of five years, and the spatial distribution characteristics of the images at each resolution during each period were analyzed. Given that each period could include imagery at different resolutions, the spatial distributions and area proportions of different imagery combinations were also analyzed for each period. Furthermore, the images at each resolution across different periods were analyzed to determine the repeated-coverage regions and corresponding time spans that can be used for land-use change detection in imagery at a specific resolution.

## 2. Materials and Methods

### 2.1. Data Preparation

The bounding vector files for full-archive Keyhole satellite images for China were downloaded from the United States Geological Survey (USGS) website, USA. The Chinese boundary was obtained from the National Geomatics Center of China. The image series were named KH-1, KH-2, KH-3, KH-4, KH-4A, KH-4B, KH-5, KH-6, KH-7, KH-9L, and KH-9H based on the characteristics of the satellite system (Table 1) [7]. Since the ground resolution of KH imagery ranges from sub-meter to ten meters, the images were classified into three resolution levels: the meter level (C1), the five-meter level (C2), and the ten-meter level (C3). The classification criteria and procedures were detailed in the reference [30]. All bounding vector files of Keyhole imagery and the Chinese national boundaries were projected by using the Krasovsky 1940 Albers projection.

A grid of points over China spaced at 10 km was generated by applying the Fishnet function in ArcMap 10.2 and used to obtain and analyze the spatial coverage distributions. The 10 km point spacing for fishnet grid generation was rigorously selected based on Keyhole imagery characteristics and sampling theory. Table 1 shows the image coverage areas, which ranged from 1111 km² (KH-7) to >4000 km² (most designators), ensuring that each image intersected >10 points (minimum for KH-7) to >40 points (typical for other systems). This spacing ensured that the grid resolution was fine enough to capture all unique spatial information given the minimum Keyhole image dimensions (~33 km for KH-7). The authors of prior studies using declassified imagery [31] similarly adopted this conservative approach, which may lead to a slight underestimation of the total coverage area, as only fully covered grid cells are counted, guaranteeing reliable change detection—a critical requirement for multi-temporal analysis. This design aligns with established practices in historical satellite studies and maintains computational efficiency for continental-scale processing.

### 2.2. Data Analysis

In this research study, we first assessed the overall spatial distribution of coverage frequency for the three resolution categories of Keyhole imagery (C1, C2, and C3) between 1960 and 1984. Then, the 25-year interval was divided into five periods to obtain detailed characteristics of the coverage frequency distribution of imagery at each resolution within each period. Given that any point generated with the Fishnet function could be covered by multi-resolution imagery within any period, the spatial distribution of images at each resolution was assessed for each period. Since land-use change analysis requires certain regions to have image coverage in multiple periods, the spatial distributions of imagery combinations among the five periods and repeated coverage across periods were obtained.

#### 2.2.1. Spatial Distribution of Coverage Frequency Between 1960 and 1984

The data analysis process (uniformly applied to all imagery categories) is demonstrated here by using C1 imagery as an illustrative example. Due to the potential overlap of multiple C1 images at each grid point, the bounding vector file of C1 imagery was spatially joined with the grid point file using ArcMAP’s Spatial Join function. The Join Operation parameter was set to JOIN_ONE_TO_ONE, and the coverage frequency of C1 imagery at each grid point was summarized. The grid points were then resampled into a raster, with each cell value being set to the coverage frequency of the corresponding point, yielding the spatial distribution of coverage frequency.

#### 2.2.2. Spatial Distribution of Coverage Frequency in Different Time Periods

The entire period from 1960 to 1984, in which declassified Keyhole imagery was available, was divided into five intervals, each covering five years [32]: T1 (1960–1964), T2 (1965–1969), T3 (1970–1974), T4 (1975–1979), and T5 (1980–1984). ArcMAP’s Spatial Join tool was employed to merge the grid point file with the bounding vector file of C1 imagery, as multiple C1 images could overlap at any grid point during various periods. The Join Operation parameter was adjusted to JOIN_ONE_TO_MANY [33], while the imagery time field in the attribute table of the image boundary was maintained. A Position–Time (PT) point set was generated as the resulting shapefile, with each PT capturing the acquisition time of a specific image at a given location and multiple PTs being possible at each grid point. All PT points within each grid point were categorized into their respective periods by referring to the start and end times of each period. The total count of PT points at a grid point in each period reflects the coverage frequency of that grid point during the corresponding time interval.

#### 2.2.3. Spatial Distribution of Imagery Combinations Across Different Time Periods

The spatial distribution of imagery at each resolution (C1, C2, and C3) varied uniquely across different time periods, highlighting that the three imagery types seldom overlapped consistently, even within the same period. Both spatial and temporal variations occurred in the imagery combination at each grid point. In this study, we quantified the spatial distribution of the imagery combinations for each period to provide a detailed understanding of the overall Keyhole imagery distribution across different periods. Eight potential combinations of the images at the three resolutions (C1, C2, and C3) were possible: C1, C2, C3, C1/C2, C2/C3, C1/C3, C1/C2/C3, and no coverage. A point file containing eight attribute fields was generated for the T1 period to document the imagery combination types. For instance, when C1 and C2 images were the only ones to provide coverage at a grid point, the “C1/C2” field was set to 1. The remaining T1 fields were assigned values by applying the same method, and point files for T2, T3, and T4 were generated, and their fields populated. The imagery combination for every grid point in each period was identified based on the imagery coverage details for various resolutions and time periods from Section 2.2.2, and the associated area proportions were computed.

#### 2.2.4. Spatial Distribution of Repeated Coverage Across Different Periods

The detection of land-use changes in a specific area using Keyhole imagery necessitates multiple-coverage data from various periods, prompting the extraction of repeated-coverage imagery at each resolution at which detection can be performed. A total of 32 combinations (2^5^ = 32), including no coverage, could be derived from the five periods of T1, T2, T3, T4, and T5: T1, T2, T3, T4, T5, T1/T2, T1/T3, T1/T4, T1/T5, T2/T3, T2/T4, T2/T5, T3/T4, T3/T5, T4/T5, T1/T2/T3, T1/T2/T4, T1/T2/T5, T1/T3/T4, T1/T3/T5, T1/T4/T5, T2/T3/T4, T2/T3/T5, T2/T4/T5, T3/T4/T5, T1/T2/T3/T4, T1/T2/T3/T5, T1/T2/T4/T5, T1/T3/T4/T5, T2/T3/T4/T5, and T1/T2/T3/T4/T5. A point file containing the 32 attribute fields mentioned above was generated for C1 imagery to document the types of period combinations. For example, if a grid point was exclusively observed during the T1, T2, and T4 periods, the T1/T2/T4 field was marked with a value of 1. The remaining fields for C1 were filled by using the same technique, and point files for C2 and C3 were similarly developed and their fields completed.

## 3. Results

### 3.1. Frequency Distribution of Total Coverage Between 1960 and 1984

**Figure 1:**
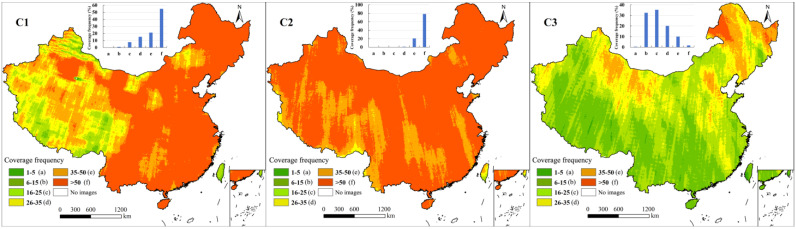
Coverage frequency spatial distribution of Keyhole imagery from 1960 to 1984 (C1, C2, and C3).

Keyhole imagery at all three resolutions covers the entire area of China, but its coverage frequencies and spatial distributions vary significantly across the different resolutions (Figure 1). The average coverage frequencies of C1, C2, and C3 imagery were found to be 58, 65, and 22 passes, respectively. Areas with a coverage frequency of fewer than 25 passes in C1 account for less than 10% of the total area, and there are no areas with a coverage frequency of fewer than 5 passes. Areas with a coverage frequency of more than 50 passes account for 55% and are mainly concentrated in eastern China. For C2 imagery, areas with coverage frequency of more than 35 and 50 passes account for 99% and 78% of China, respectively. There are almost no areas with coverage frequencies of fewer than five passes or more than 50 passes for C3. Areas with a coverage frequency of more than 35 passes are mainly distributed in the Beijing–Tianjin region, western Inner Mongolia, and northeastern China. Moreover, areas with lower coverage (fewer than 15 passes) are primarily located in southwestern China.

Considering the distributions of high and low values for imagery at each resolution, the coverage is relatively lower for southwestern China and Taiwan, and higher coverage was observed in northeastern China, central Xinjiang, and western Inner Mongolia. The difference in the priority of the United States’ military surveillance of Chinese regions could be the main reason for the uneven coverage frequency distribution. Taiwan and the United States shared close relations, and this region might not have been considered a priority for military surveillance, which could account for the lower coverage frequency [34]. The higher coverage frequency in the northeast was likely due to the numerous military–industrial facilities built with Soviet assistance in the 1950s [35]. Additionally, extensive nuclear exploration was carried out at Chinese northwestern test sites from the 1960s to the 1980s [36].

### 3.2. Frequency Distribution of Coverage in Five 5-Year Periods

#### 3.2.1. C1 Imagery

**Figure 2:**
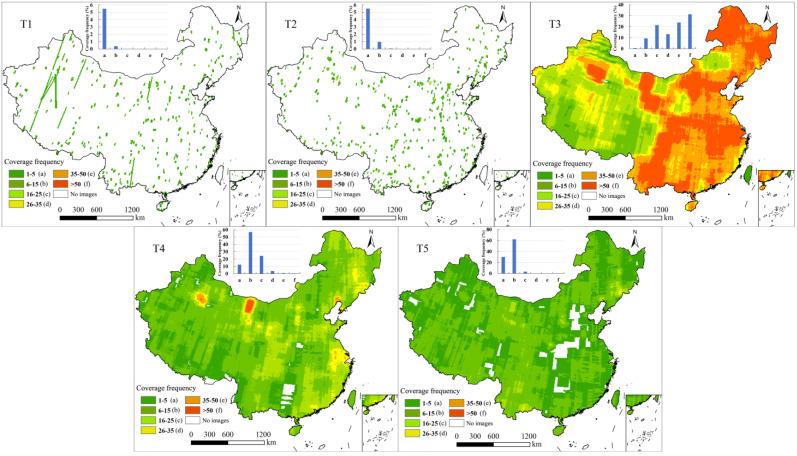
Coverage frequency spatial distribution of C1 imagery in different periods.

Although C1 images were distributed across all five periods, the spatial characteristics of their coverage area and frequency varied significantly among different periods. The coverage rate in both the T1 and T2 periods was only 6% (Figure 2). The coverage frequency in all areas was fewer than five passes and exhibited a scattered distribution pattern. The entire region of China was covered by Keyhole imagery during the T3 period, with an average coverage frequency of 40 passes. Areas with a coverage frequency of more than 50 and 15 passes accounted for 50% and 90% of the total, respectively. The distribution pattern of high coverage for the T3 period was similar to that of all C1 imagery, concentrated in eastern China, western Inner Mongolia, and northern Xinjiang. Low-coverage areas were concentrated in XiZang and southwestern China. The average coverage frequency for the T4 period was 12 passes, with most areas falling within the 6~15 and 16~25 ranges, accounting for 57% and 24% of the total, respectively. Areas with more than 50 passes were concentrated in western Inner Mongolia and northern Xinjiang, and regions with low coverage frequencies were primarily located in southwestern China. Although the percentage of coverage area for the T5 period was similar to that for the T4 period, the average coverage frequency was only 6.6 passes. The coverage frequency was mainly concentrated in the range of 1~5 and 6~15 passes, with area proportions of 30% and 62%, respectively. Low coverage frequencies were found for regions mainly distributed in central China and differed from those in southwest China during the T3 and T4 periods.

#### 3.2.2. C2 Imagery

**Figure 3:**
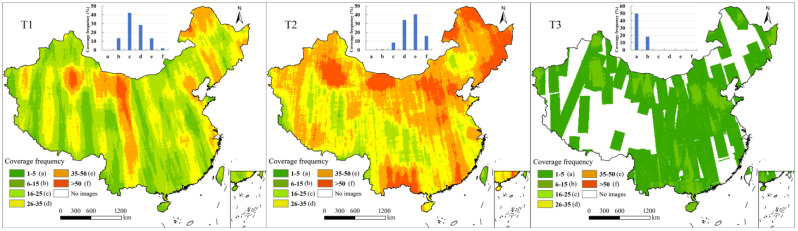
Coverage frequency spatial distribution of C2 imagery in different periods.

The C2 images were only distributed in the T1, T2, and T3 periods. The coverage rates in both T1 and T2 were 100%, with average coverage frequencies of 25 and 38 passes, respectively (Figure 3). In the T1 period, the area with a coverage frequency of fewer than 15 passes accounted for 14% and was distributed across different regions in China. Areas with a coverage frequency of more than 50 passes were mainly located in western Inner Mongolia, accounting for 2% of the total. Almost all areas in the T2 period had a coverage frequency exceeding 15 passes. Areas with a coverage frequency of more than 50 passes were mainly located in western Inner Mongolia, the Beijing–Tianjin region, and the northeastern Chinese border. The coverage area and frequency in the T3 period were lower than those in T1 and T2, with values of only 68% and four passes, respectively. The coverage frequency in 50% of the total area was fewer than five passes, and areas without imagery were mainly located in the western and central–northeastern regions of China.

#### 3.2.3. C3 Imagery

**Figure 4:**
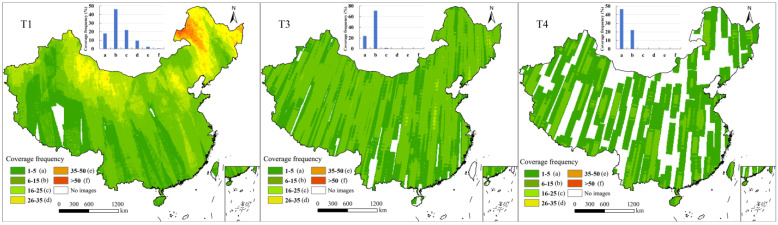
Coverage frequency spatial distribution of C3 imagery in different periods.

C3 imagery was available in the T1, T3, and T4 periods (Figure 4). The coverage rates in the T1 and T3 periods were relatively high, 98% and 96%, respectively, with average coverage frequencies of 13 and seven passes, respectively. In the T1 period, 86% of the total area had a coverage frequency of fewer than 25 passes. Areas with a coverage frequency of more than 35 passes were primarily located along the western border of northeastern China. The coverage frequency in the T2 period was concentrated between 1~5 and 6~15 passes, with area proportions of 24% and 71%, respectively. The coverage rate in the T3 period was 69%, with an average coverage frequency of four passes. The coverage frequency was concentrated between 1~5 and 6~15 passes, with area proportions of 47% and 22%, respectively.

### 3.3. Distribution of Imagery Combinations Across Five 5-Year Periods

**Figure 5:**
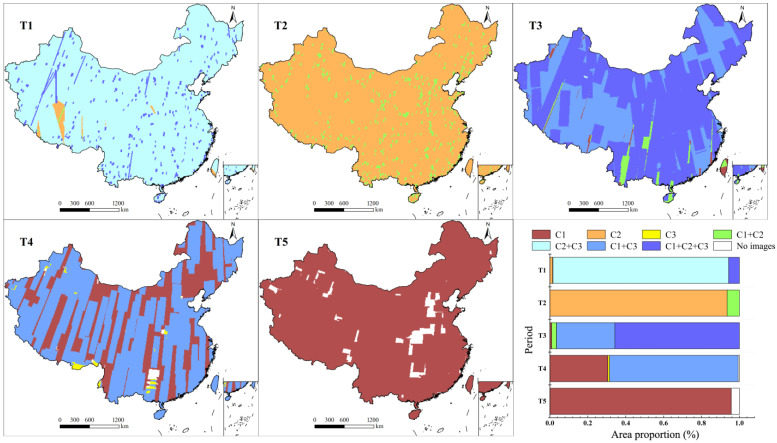
Spatial distributions and proportions of coverage of imagery combinations across five 5-year periods.

The imagery combination distributions reflected the types of images available for a given area. The coverage proportions of C2 and C3 imagery for the T1 period were 100% and 98%, respectively, while that of C1 imagery was only 6% (Figure 5). As a result, the combination of C2/C3 accounted for 93% of the total area in the T1 period, and only 6% was covered by C1/C2/C3. This means that most land use in T1 could be captured by using both C2 and C3 images, while less than 10% of the region was covered by imagery at all three resolutions. The T3 period also featured coverage provided by imagery at all three resolutions. However, the distributions of imagery combinations during this period were more complex than those in T1. More than half of the total area (66%) was covered by imagery at all three resolutions in T3. Areas covered by the C1/C3 imagery combination accounted for 31% of the total area and were primarily located at Xizhang and in northeastern China. Compared with T1, which also had full-resolution coverage, the larger-area coverage provided by C1, C2, and C3 simultaneously was mainly due to the greater coverage of imagery at each resolution, i.e., 100%, 68%, and 96%, respectively. For the T2 period, there was a significant difference in coverage between C1 and C2 images, 7% and 100%, respectively. As a result, most land use in T2 could only be captured by C2 imagery. A total of 7% of areas were covered by the C1/C2 imagery combination, whose distribution was similar to that of C1 imagery in this period. During the T4 period, areas covered by both C1 and C3 accounted for 68% of China, and the remaining areas were only covered by C1 imagery. For the T5 period, only C3 imagery was available, covering 96% of the total area.

### 3.4. Repeat-Coverage Distribution Across Periods

**Figure 6:**
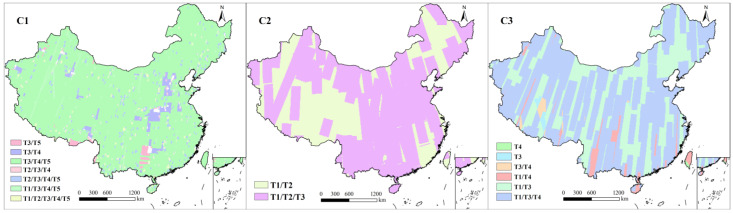
Spatial distribution of repeated coverage in different periods.

Although imagery at a certain resolution is available for all periods, the coverage proportion differences among different periods affect the regions, periods, and proportions based on which land-use detection can be conducted. The coverage proportions of C1 were relatively high in the T3, T4, and T5 periods, 100%, 98%, and 99%, respectively (Figure 6). As a result, the areas for which land-use change could be detected across T3, T4, and T5 accounted for 94% of the total area, meaning that two instances of land-use change, from T3 to T4 and from T4 to T5, could be captured by using C1 imagery. Since the coverage proportion of C1 during T1 and T2 was only 6%, only 2% of the total area allowed for land-use change detection across the five periods. The coverage of C2 during the T1 and T2 periods accounted for 100%, meaning that land-use change during these two periods could be detected across the whole of China. The area proportion of T1/T2/T3 was 68%, but only one-event land-use changes could be obtained for regions including Xizang and the northeastern and southern coastal areas. Due to the higher coverage of C3 imagery in the T1 and T3 periods (98% and 96%, respectively) compared with T4 (69%), the area proportion of the time spans associated with the T4 period was smaller than that excluding T4. Almost all areas (95%) allowed for the acquisition of land-use change between the T1 and T3 periods, while the corresponding proportions for T3/T4 and T1/T4 were only 65% and 68%, respectively. Two-event land-use changes across T1/T3/T4 could be captured in more than half of the total area (64%).

### 3.5. Application of Paid Keyhole Images Selection for Multiple Periods

**Figure 7:**
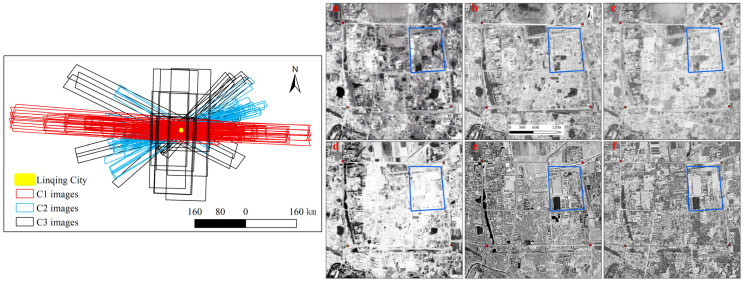
Multi-temporal and multi-resolution paid Keyhole images for land-use change detection (E 115.731 N 36.845). (**Left**) Distribution of paid images; (**Right**) free images showing land-use change. (**a**) 30 August 1961. (**b**) 23 August 1965. (**c**) 19 August 1966. (**d**) 20 September 1967. (**e**) 20 March 1973. (**f**) 19 August 1979. The blue box in each graph showed landuse change across periods.

Here, we select Linqing City in Shandong Province (E 115.731 N 36.845) to demonstrate the potential of Keyhole imagery for the detection of land-use changes (Figure 7). The left figure shows the distribution of paid Keyhole images at three resolutions covering Linqing City, and the right one displays the land-use change within the time-series free images. Since the free imagery already provided multi-temporal and multi-resolution coverage, and the paid imagery was not freely accessible, we applied free imagery to demonstrate land-use change details in this research study, while paid imagery was used to illustrate spatial coverage and multi-temporal potential. The acquisition times from (a) to (f) in the right figure are 30 August 1961, 23 August 1965, 19 August 1966, 20 September 1967, 20 March 1973, and 19 August 1979. The four red points indicate the old town of Linqing city, constructed around the year 1767, and the blue rectangle shows areas with clear land-use change across the above periods.

Table 2 presents the number and corresponding acquisition times of paid and free Keyhole images in different periods and at different resolutions for Linqing City. The total number of paid images (165) was larger than that of free images (24) at all three resolutions, and the number of paid images in each period was larger than that of free images. The proportions of different resolutions varied significantly between the two datasets. High-resolution C1 images accounted for over 40% of the paid imagery, whereas in the free imagery, they constituted only 21%. The temporal coverage of the free imagery was also limited, as it was missing certain periods covered by the paid data, such as T5 for C1, T1 for C2, and T4 for C3. Therefore, paid imagery potentially provides broader applicability for land-use change analysis than free imagery. The acquisition period of paid images starts earlier than or at the same time as that of free images and ends later than or at the same time as that of free imagery. As a result, the time span of the paid images was longer or the same as that of the free images. Specifically, the time span of both C1 and C2 paid images was longer than that of the free images, and only C3’s time span was the same in both datasets.

## 4. Discussion

### 4.1. Potential of Declassified Imagery for Extending Historical Land-Use Change Research

Land-use changes should not be conceived as unidirectional developments following predefined trajectories but rather as path-dependent processes [37] that may be affected by various drivers, including sudden events. Understanding past land-use changes and driving forces can lead to a better understanding of current land-use trends and provide a scientific basis for predicting future changes [38]. The development of current remote sensing technologies and methods has already made a significant impact on the monitoring of land-cover and land-use changes at a variety of scales. Since historical aerial imagery is almost the only data type that can provide a direct map view of past land-use patterns, it is thought that it will become increasingly critical in the future as more historical data are declassified. The imagery featured in this study included both free and pay-to-download declassified Keyhole data from 1960 to 1984. According to the metadata of the archived Keyhole imagery of the USGS, the number of paid images covering China was approximately 16 times that of the free images. Keyhole imagery paid data were priced at USD 30 per scene [31], with costs of USD 3.8 and USD 6.0 per 1 k square kilometer for meter-level and five-meter-level images, respectively, which were significantly lower than historical aerial imagery costs. Therefore, Keyhole imagery could provide significant data support for extending the study period of existing studies on terrestrial landscape change and related research. U2 aerial imagery (1950~1960) is another important data source for historical land-use detection. Although the coverage area of all U2 missions is not global as in the Keyhole missions, the ground resolution of U2 (about 1 m) [39] is much higher than that of most Keyhole imagery, except KH-7 and KH-9H.

Among the various historical data sources, the global coverage of Keyhole imagery gives it a relatively promising application prospect. Aerial imagery or historical topographic maps typically only cover specific areas of interest, with almost no datasets being available for large-scale or national-level spatial continuity. Variations in the resolution and accuracy of imagery (map) data make it difficult to perform precise comparisons of research results on land-use change across different regions [40]. In this study, we demonstrated that the repeated coverage of Keyhole imagery allows for the capture of land surface changes from the 1960s to the 1980s. For C1, C2, and C3, at least one land-use change event could be detected in more than 90% of the total area of China during the 1960s and 1980s, although the periods varied for different images. Similarly, the areas where at least one land-use change event between two periods in the 1960s and 1980s could be identified were 94%, 68%, and 64% of the total area for C1, C2, and C3, respectively. Previous research shows that free Keyhole images provided around 30% of national coverage for one-event land-use changes and only 7.2% of national coverage for two-event land-use changes, while paid images extend this capability to 94% of the country. The paid collection also significantly improves resolution-specific utility, increasing C1’s land-use change detection applicability from 26.5% to 94% of the study area and boosting C2’s multi-period analysis potential from 10% to 68%. A key limitation is that the area proportions for land-use change detection in this research study are not generalizable, as Keyhole imagery’s observation priority differs across regions (countries). Nonetheless, other regions globally can also employ the methodology for the repeated-coverage distribution analysis of Keyhole imagery developed in this research study. Additionally, researchers can leverage our Python (3.12) code to efficiently analyze the spatiotemporal distribution of Keyhole imagery and other historical data with comparable metadata in their regions of interest.

### 4.2. Restrictions of Keyhole Imagery and Other Historical Data

The extensive use of Keyhole images is restricted by the complexities of panoramic imaging geometry, film distortions, and the limited availability of georeferencing data [41]. The main challenge in employing Keyhole imagery is the scarcity of sufficient ground control points for image correction, as terrestrial landscapes have drastically changed from the 1960s and 1970s to the present. The absence of cloud-cover indicators in the USGS metadata for Keyhole imagery makes cloud detection essential to the application of high-resolution remote sensing imagery. Over two-thirds of the Earth’s surface is continuously covered by clouds [42], and Landsat 5 and 7 images can feature up to 40% of cloud cover [43]. Hence, cloud cover may interfere with the detection of land surface changes when analyzing multi-temporal imagery. Additionally, spectral information-dependent land-use classification methods frequently fail to achieve high accuracy due to the sparse spectral information in Keyhole’s single-band images [44].

The broad utilization of other historical graphical data, similar to Keyhole imagery, is confronted with numerous technical obstacles. Transforming non-standardized historical datasets into digitized and standardized formats often entails significant time and financial investment. Although U2 is publicly available, these images are not accessible since the imagery films have not been digitized, or even printed [45]. Researchers need to autonomously work out how images are organized to access them, and such a tedious process has undoubtedly hindered historical data applications. Of the 700,000 restored map sheets in the U.S. Library of Congress, which cover 12,000 cities and towns in the U.S., Canada, Cuba, and Mexico, only 25,000 sheets for more than 3000 cities are available as scanned documents [46]. In a similar effort, historical aerial photographs and maps from foreign collections, especially in Europe and North America, were analyzed by Polish researchers in a cultural heritage study [47]. The lack of detailed metadata for these collections poses a significant barrier to their utilization. Historical maps are a vital resource for studying past environments in the era before aerial photography and satellite imagery existed. Processing and extracting information from historical maps are greatly complicated by the differing symbologies resulting from changes in cartographic design practices. Consequently, the complete utilization of historical graphical data requires the combined efforts of experts from various fields.

## 5. Conclusions

This study demonstrates the emerging potential of declassified Keyhole (KH) satellite imagery for the reconstruction of multi-temporal land-use changes during the 1960–1984 period, bridging a critical gap in pre-Landsat-era Earth observation. Our automated analysis of 300,000+ image footprints reveals distinct spatiotemporal patterns with direct policy relevance: meter-resolution (C1) imagery provides near-complete coverage (94%) for monitoring post-1970 urban expansion and deforestation trends, while earlier five-meter (C2) datasets capture land-use baselines in the 1960s, which are critical for validating historical territory claims and environmental compensation cases. These findings equip policymakers with spatially explicit evidence to support land restoration initiatives and settle long-standing disputes where documentary records are scarce.

The Python pipeline allows for the efficient processing of metadata to identify optimal change detection periods—revealing, for instance, that for 64% of the Chinese territory, two-phase land transitions (1960s–1970s–1980s) can be tracked with combined multi-resolution analysis. This capability is particularly valuable for assessing path-dependent environmental changes, such as reforestation in former agricultural areas or wetland degradation trajectories.

Limitations (e.g., single-band spectral constraints and cloud-cover uncertainty) highlight opportunities to integrate KH with ancillary datasets (e.g., declassified maps and U-2 imagery). Future work should prioritize machine learning techniques to extract land-use information from KH’s panchromatic data, further unlocking its value for historical ecology and policy design.

## Figures and Tables

**Table 1:** Metadata of full-archive Keyhole imagery for China.

Satellite	Resolution (Feet/m)	Number of Images	Period Start and End	Singe Area (km^2^)	Resolution Category	Total Number
KH-7	(2 to 4)/(0.9)	2693	July 1963~June 1967	1100 ± 848	C1	106,824
KH-9H	(2 to 4)/(0.9)	104,231	March 1973~October 1980	6222 ± 4854		
KH-4B	6/1.8	42,413	September 1967~May 1972	4048 ± 855	C2	152,129
KH-4A	9/2.8	109,716	August 1963~September 1969	5137 ± 703		
KH-9L	(20 to 30)/7.6	6813	June 1971~October 1984	29,133 ± 2012	C3	21,685
KH-3	25/7.6	1911	August 1961~December 1961	9396 ± 1272		
KH-4	25/7.6	12,307	February 1962~December 1963	10,524 ± 7053		
KH-2	30/9.1	654	December 1960~July 1961	11,707 ± 5187		
KH-1	40/12.2	30	August 1960	9613 ± 334	Not accounted	533
KH-6	6/1.8	44	July 1963~August 1963	10,524 ± 7053		
KH-5	460/153.3	459	February 1961~August 1964	337,664 ± 45,073		

**Table 2:** Paid and free Keyhole images covering Linqing City (E 115.731 N 36.845).

Datasets	Resolution	Total Image Number	Image Number per Period					Start Date	End Date
			T1	T2	T3	T4	T5		
Paid	C1	68	0	0	39	22	7	20 June 1971	4 February 1981
	C2	69	13	44	12	0	0	17 February 1964	27 September 1971
	C3	28	18	0	0	7	3	30 August 1961	2 September 1980
Free	C1	5	0	0	2	3	0	20 June 1971	19 August 1979
	C2	14	0	10	4	0	0	23 August 1965	22 November 1970
	C3	5	2	0	0	0	2	30 August 1961	2 September 1980

## Data Availability

The original datasets analyzed during the current study are available from the United States Geological Survey website (https://earthexplorer.usgs.gov/ (accessed on 4 March 2024)). The source code and downloaded data that support the findings in this study are available at https://doi.org/10.6084/m9.figshare.28667549.

## References

[1] Zhu Z., Woodcock C.E. (2014). Continuous change detection and classification of land cover using all available Landsat data. Remote Sens. Environ..

[2] Wang M., Wander M., Mueller S., Martin N., Dunn J.B. (2022). Evaluation of survey and remote sensing data products used to estimate land use change in the United States: Evolving issues and emerging opportunities. Environ. Sci. Policy.

[3] Stanimirova R., Tarrio K., Turlej K., McAvoy K., Stonebrook S., Hu K.-T., Arévalo P., Bullock E.L., Zhang Y., Woodcock C.E. (2023). A global land cover training dataset from 1984 to 2020. Sci. Data.

[4] Wu B., Fu Z., Fu B., Yan C., Zeng H., Zhao W. (2024). Dynamics of land cover changes and driving forces in China’s drylands since the 1970 s. Land Use Policy.

[5] Dashora A., Lohani B., Malik J. (2007). A repository of earth resource information CORONA satellite programme. Curr. Sci..

[6] Richelson J. (1984). The keyhole satellite program. J. Strateg. Stud..

[7] Fowler M.J.F., Hanson W.S., Oltean I.A. (2013). Declassified Intelligence Satellite Photographs. Archaeology from Historical Aerial and Satellite Archives.

[8] Di Giacomo G., Scardozzi G. (2012). Multitemporal High-Resolution Satellite Images for the Study and Monitoring of an Ancient Mesopotamian City and its Surrounding Landscape: The Case of Ur. Int. J. Geophys..

[9] Shahbandeh M., Kaim D., Kozak J. (2023). Using CORONA Imagery to Study Land Use and Land Cover Change—A Review of Applications. Remote Sens..

[10] Zhang Y., Shen W., Li M., Lv Y. (2020). Integrating Landsat Time Series Observations and Corona Images to Characterize Forest Change Patterns in a Mining Region of Nanjing, Eastern China from 1967 to 2019. Remote Sens..

[11] Song D.-X., Huang C., Sexton J.O., Channan S., Feng M., Townshend J.R. (2015). Use of Landsat and Corona data for mapping forest cover change from the mid-1960s to 2000s: Case studies from the Eastern United States and Central Brazil. ISPRS J. Photogramm. Remote Sens..

[12] Fowler M.J.F. (2006). Archaeology through the keyhole: The serendipity effect of aerial reconnaissance revisited. Interdiscip. Sci. Rev..

[13] Shangguan D.H., Bolch T., Ding Y.J., Kröhnert M., Pieczonka T., Wetzel H.U., Liu S.Y. (2015). Mass changes of Southern and Northern Inylchek Glacier, Central Tian Shan, Kyrgyzstan, during ~1975 and 2007 derived from remote sensing data. Cryosphere.

[14] Makovics J.L. Utilising Declassified Cold War Satellite Imagery (KH-9 Hexagon) for Remote Sensing of Historic Hydraulic Management Features in North Africa. Proceedings of the 2024 IEEE Mediterranean and Middle-East Geoscience and Remote Sensing Symposium (M2GARSS).

[15] Brecheisen Z., Hamp-Adams N., Tomasek A., Foster E.J., Filley T., Soto M.V., Reynoso L.Z., de Lima Moraes A., Schulze D.G. (2020). Using Remote Sensing to Discover Historic Context of Human-Environmental Water Resource Dynamics. J. Contemp. Water Res. Educ..

[16] Chang A.Y., Parrales M.E., Jimenez J., Sobieszczyk M.E., Hammer S.M., Copenhaver D.J., Kulkarni R.P. (2009). Combining Google Earth and GIS mapping technologies in a dengue surveillance system for developing countries. Int. J. Health Geogr..

[17] Winkler K., Fuchs R., Rounsevell M., Herold M. (2021). Global land use changes are four times greater than previously estimated. Nat. Commun..

[18] Lasaponara R., Yang R., Chen F., Li X., Masini N. (2018). Corona Satellite Pictures for Archaeological Studies: A Review and Application to the Lost Forbidden City of the Han–Wei Dynasties. Surv. Geophys..

[19] Nistor C., Vîrghileanu M., Cârlan I., Mihai B.-A., Toma L., Olariu B. (2021). Remote Sensing-Based Analysis of Urban Landscape Change in the City of Bucharest, Romania. Remote Sens..

[20] Guo Z., Ye Q., Li F., Wang Y.J.I.T.o.D., Insulation E. (2019). Study on corona discharge spatial structure and stages division based on visible digital image colorimetry information. IEEE Trans. Dielectr. Electr. Insul..

[21] Agapiou A., Alexakis D.D., Sarris A., Hadjimitsis D.G. (2016). Colour to Greyscale Pixels: Re-seeing Greyscale Archived Aerial Photographs and Declassified Satellite CORONA Images Based on Image Fusion Techniques. Archaeol. Prospect..

[22] Agapiou A. (2021). Land Cover Mapping from Colorized CORONA Archived Greyscale Satellite Data and Feature Extraction Classification. Land.

[23] Stratoulias D., Grekousis G. (2021). Information Extraction and Population Estimates of Settlements from Historic Corona Satellite Imagery in the 1960s. Sensors.

[24] Hamandawana H., Eckardt F., Ringrose S. (2007). Proposed methodology for georeferencing and mosaicking Corona photographs. Int. J. Remote Sens..

[25] Darack E. (2019). Spying from above: How the atmosphere helped shape our modern surveillance systems. Weatherwise.

[26] Brown D.G., Johnson K.M., Loveland T.R., Theobald D.M. (2005). Rural land-use trends in the conterminous United States, 1950–2000. Ecol. Appl..

[27] Ramankutty N., Graumlich L., Achard F., Alves D., Chhabra A., DeFries R.S., Foley J.A., Geist H., Houghton R.A., Goldewijk K.K., Lambin E.F., Geist H. (2006). Global Land-Cover Change: Recent Progress, Remaining Challenges. Land-Use and Land-Cover Change: Local Processes and Global Impacts.

[28] Pontius R.G., Roopa K., Laura S., Yan Y., Zhang Y. (2017). Methods to summarize change among land categories across time intervals. J. Land Use Sci..

[29] Li H., Yao W., Zhang M., Yang X., Wang Q. (2025). Spatial heterogeneity of keyhole imagery coverage in China and imagery dataset cost estimation. Sci. Rep..

[30] Li H., Wang T., Yao W., Liu H., Song C., Sun J. (2025). Multitemporal Analysis of Declassified Keyhole Imagery’ for Landuse Change Detection in China (1960~1984): A Python-Based Spatial Coverage and Automation Workflow. Remote Sens..

[31] Casana J. (2020). Global-Scale Archaeological Prospection using CORONA Satellite Imagery: Automated, Crowd-Sourced, and Expert-led Approaches. J. Field Archaeol..

[32] Yang S., Zhao W., Liu Y., Wang S., Wang J., Zhai R. (2018). Influence of land use change on the ecosystem service trade-offs in the ecological restoration area: Dynamics and scenarios in the Yanhe watershed, China. Sci. Total Environ..

[33] Crowther M.S., Lunney D., Lemon J., Stalenberg E., Wheeler R., Madani G., Ross K.A., Ellis M. (2014). Climate-mediated habitat selection in an arboreal folivore. Ecography.

[34] Abidde S.O., Abidde S.O. (2022). The US-China-Taiwan Relations: Military Invasion, Annexation, and Verbal Brinkmanship. China and Taiwan in Africa: The Struggle for Diplomatic Recognition and Hegemony.

[35] Gobarev V.M. (1999). Soviet policy toward China: Developing nuclear weapons 1949–1969. J. Slav. Mil. Stud..

[36] Babiarz R. (2015). The People’s Nuclear Weapon: Strategic Culture and the Development of China’s Nuclear Weapons Program. Comp. Strategy.

[37] Chavez A.B., Perz S.G. (2013). Path dependency and contingent causation in policy adoption and land use plans: The case of Southeastern Peru. Geoforum.

[38] Lambin E.F., Rounsevell M.D.A., Geist H.J. (2000). Are agricultural land-use models able to predict changes in land-use intensity?. Agric. Ecosyst. Environ..

[39] Li Z., Mengdi Z., Yukun Z., Li Y. (2023). Automatic detection of beacon towers in historical aerial images using improved FCOS. Int. J. Remote Sens..

[40] Balha A., Mallick J., Pandey S., Gupta S., Singh C.K. (2021). A comparative analysis of different pixel and object-based classification algorithms using multi-source high spatial resolution satellite data for LULC mapping. Earth Sci. Inform..

[41] Ghuffar S., Bolch T., Rupnik E., Bhattacharya A. (2022). A Pipeline for Automated Processing of Declassified Corona KH-4 (1962–1972) Stereo Imagery. IEEE Trans. Geosci. Remote Sens..

[42] Mbatha N., Shikwambana L. (2022). First Observations of Cirrus Clouds Using the UZ Mie Lidar over uMhlathuze City, South Africa. Appl. Sci..

[43] Feng L., Wang X. (2024). Quantifying Cloud-Free Observations from Landsat Missions: Implications for Water Environment Analysis. J. Remote Sens..

[44] Deshpande P., Anirudh B., Onkar D., Tripathi S. (2021). Historical land cover classification from CORONA imagery using convolutional neural networks and geometric moments. Int. J. Remote Sens..

[45] Hammer E., Lauricella A. (2017). Historical Imagery of Desert Kites in Eastern Jordan. Near East. Archaeol..

[46] Uhl J.H., Leyk S., Chiang Y.-Y., Knoblock C.A. (2022). Towards the automated large-scale reconstruction of past road networks from historical maps. Comput. Environ. Urban Syst..

[47] Janus J., Bożek P., Taszakowski J., Doroż A. (2022). Decaying villages in the centre of Europe with no population decline: Long-term analysis using historical aerial images and remote sensing data. Habitat Int..

